# Update on the diagnosis and management of exocrine pancreatic insufficiency

**DOI:** 10.12688/f1000research.20779.1

**Published:** 2019-11-26

**Authors:** Yaseen Perbtani, Chris E. Forsmark

**Affiliations:** 1 Division of Gastroenterology, Hepatology, and Nutrition, University of Florida, Gainesville, FL, USA

**Keywords:** Exocrine Pancreatic Insufficiency, pancreatic enzyme replacement therapy, chronic pancreatitis

## Abstract

Exocrine pancreatic insufficiency (EPI) is characterized by inadequate pancreatic enzyme delivery to the small intestine Exocrine pancreatic insufficiency (EPI) is characterized by inadequate pancreatic enzyme delivery to the small intestine, resulting in malabsorption. Clinical manifestations of EPI are often nonspecific and can lead to lack of timely recognition and diagnosis. Central to this clinical dilemma is the lack of highly accurate or specific testing which leads to misdiagnosis and suboptimal treatment. Identification of high-risk patients is key in the diagnosis of EPI and this includes patients with pancreatic parenchyma disorders such as chronic pancreatitis, pancreatic malignancy, cystic fibrosis, and those undergoing pancreatic resection for benign and malignant disease. Less recognized are the number of additional conditions which may also have EPI as a consequence. Owing to an increase in morbidity and impaired quality of life associated with this condition, goals of treatment have been aimed at repleting exocrine enzyme deficiency by oral pancreatic enzyme replacement therapy (PERT). The basis of PERT is to provide activated digestive enzymes to the small bowel during the prandial period, mainly, leading to sufficient absorption of fat and fat-soluble vitamins. The benefits of PERT have been shown to go beyond the improvement in signs and symptoms associated with EPI and include decreasing prevalence of osteopathy and improving survival outcomes in subsets of patients with this condition. However, despite the overall benefits in treatment, the diagnosis and management of EPI are suboptimal. Current literature suggests patients at high risk of developing EPI are not tested and those who are diagnosed are not treated with adequate dosages. In this review, we highlight patients who are at high risk for the development of EPI, analyze consequences and treatment of this disorder, review rationale for enzyme replacement therapy, and examine current evidence for treatment optimization.

## Introduction

Exocrine pancreatic insufficiency (EPI) is characterized by the maldigestion of macronutrients and micronutrients as a result of inadequate intraduodenal pancreatic exocrine enzyme delivery. Manifestations of EPI include steatorrhea, malnutrition, trace element and vitamin deficiency, abdominal discomfort, bloating, and metabolic bone disease, which ultimately can lead to a significant decrease in quality of life ^1, 2^. The symptoms and manifestations of EPI are not specific and are shared with other common gastrointestinal conditions. This can lead to a lack of recognition and a lack of diagnostic testing in many patients. Even in those who are correctly diagnosed, many are not treated with appropriate dosages of pancreatic enzyme replacement therapy (PERT).

One central challenge in accurate diagnosis is the lack of a highly accurate diagnostic test for EPI. The diagnostic tests that are available are generally difficult to perform, inaccurate, or non-specific. Diagnostic testing can be directed at documenting the presence of maldigestion in general or at measuring pancreatic enzyme output directly or indirectly. The gold standard for diagnosing maldigestion, or malabsorption, has been 72-hour fecal fat testing. In this study, patients ingest a high-fat diet (of known fat content) for 5 days, with collection of stool output over the last 3 days. In the normal state, on a 100 g fat/day diet, at least 93% of dietary fat is absorbed ^3^. This test is difficult or impossible in a routine clinical setting, given that it is heavily reliant on accurate recording of fat intake and stool collection over 3 days. Additionally, given this test signifies only malabsorption of fat, it does not isolate the pancreas as the cause of the abnormality. Another way to diagnose EPI is by administering some type of test meal which requires digestion by pancreatic enzymes and measuring the presence of metabolites of the test substance. The usage of these tests is not common in the US (Lundh test meal); however, they are still utilized at several sites throughout Europe. Breath tests to measure fat digestion by pancreatic lipase have a role as an alternative in the diagnosis of EPI ^4– 6^; however, they are not widely available and accuracy seems to be similar to the testing of fecal elastase ^7^. Direct quantification of pancreatic ductal secretion of bicarbonate after secretin stimulation is available, but this test does not measure digestive enzyme secretion from acinar cells. Measuring enzyme output with duodenal tubes, after stimulation with the hormone cholecystokinin, is not currently performed in the US.

Unfortunately, the only currently available test for EPI is the measurement of elastase levels in stool samples ^8^. The test is performed on a single solid or semi-solid stool sample. The test, although called fecal elastase-1, does not actually measure elastase-1, as this is transcriptionally silenced in humans ^9, 10^. The test actually measures chymotrypsin-like elastases (CELA) 3A and 3B. Other pancreatic enzyme levels (chymotrypsin) can also be measured in stool, but these are not as stable during intestinal passage and are far less accurate. The advantages of fecal elastase measurement include lack of requirement of timed stool collection or a prerequisite for specific diet prior to testing. Moreover, it is widely accessible and can be performed while a patient is taking PERT (the assay measures human CELA3A and 3B and does not cross-react with porcine replacement products). The accuracy of the test depends on the chosen cut-off. It has reasonable sensitivity in advanced or severe EPI if a cut-off of <100 mcg/g of stool is chosen. Levels above 200 mcg/g stool are normal, and levels between 100 and 200 are indeterminate ^11^. Many shortcomings of this test exist and include high false positive rates dependent on cut-off levels chosen, stool consistency at the time of sampling (may be falsely low in watery stool because of dilution), and low fecal elastase in advanced age and other medical conditions such as chronic kidney disease or diabetes mellitus (DM). Therefore, the diagnosis of EPI is often heavily reliant on the identification of patients who are at increased risk and who present with suggestive clinical symptoms.

While chronic pancreatitis (CP) is the most well-known cause of EPI, other common etiologies include cystic fibrosis, pancreatic cancer, and pancreatic resection for benign or malignant disease. Less commonly known are a number of additional conditions which may also have EPI as a consequence ^12^. Because of an increase in morbidity associated with this condition, and mortality correlated with malnutrition ^13^, therapy has been targeted to replace or augment underlying exocrine enzyme deficiency by oral PERT. The principles of PERT are to supply a level of enzyme sufficient to aid in adequate digestion and absorption of fat and fat-soluble vitamins, leading to improvement in signs and symptoms associated with EPI ^14^. However, given the complexity associated with optimal dosing of PERT, due to variability in both an individual’s meal fat content and residual pancreatic function, there still remains inconsistency amongst medical professionals over optimal administration and potential benefit. The aim of this review is to identify patients who are at high risk for the development of EPI, analyze symptoms and consequences of this syndrome, review rationale for enzyme replacement, and examine evidence-based protocols for treatment optimization.

## Patients at risk for exocrine pancreatic insufficiency

### Chronic pancreatitis

CP is a disorder characterized by ongoing pathological pancreatic inflammation, leading to fibrosis and eventually to the loss of both endocrine and exocrine function. EPI most commonly develops after 5–10 years of CP, requiring approximately 90% of the endogenous pancreatic enzyme secretion to be lost ^15^. The risk of EPI varies with the underlying etiology of CP, with the highest risk population being those with chronic alcohol use ^16^. Smoking has an independent effect on the development of CP, and the probability of EPI increases when tobacco exposure is combined with alcohol ^17, 18^. Likewise, abstinence from tobacco, and alcohol, slows the development of CP and its associated complications. Certain genetic causes of CP (PRSS1 mutations causing hereditary pancreatitis) are also commonly associated with EPI. In addition, the timing of onset of EPI is closely correlated with etiology. In a natural history study by Layer *et al*. ^19^, patients with CP due to chronic alcohol use developed EPI at a faster rate (median 13.1 years) when compared to patients with early onset idiopathic CP (median 26.3 years). Notably, in all patients, and irrespective of etiology of CP, the onset of EPI may occur before, after, or at the same time as the development of endocrine insufficiency (diabetes) due to CP ^20^.

### Acute pancreatitis

EPI following severe acute pancreatitis (AP) can occur as a result of loss of pancreatic parenchyma and functional capacity due to significant amounts of pancreatic necrosis. During even less severe AP, EPI may also develop because of both reduction of enzyme release from acinar cells and ductal outlet obstruction. Additionally, pancreatic enzyme release can be impaired by alterations in hormonal mediators or disruption of downstream cell signaling responsible for stimulating enzyme production ^21^. The occurrence of EPI after AP has recently been evaluated in a meta-analysis by Huang *et al*. ^22^, who found that the cumulative prevalence was 62% at any time point between index hospitalization and follow-up. Although this drops over time, during long-term follow-up, EPI persists in up to a third of patients. This finding was congruent with a prior meta-analysis evaluating EPI following AP during a 36-month follow-up, which reported a prevalence of 27% ^23^. Although estimating the true prevalence of EPI is difficult owing to heterogeneity between relevant studies, these two large-scale analyses suggest that nearly a third of patients will develop persistent EPI after an episode of AP. Of particular interest, the development of significant pancreatic necrosis is not required for EPI to develop after AP.

### Pancreatic resection and surgery

The advancement in operative techniques and postoperative outcomes after pancreatic resection for malignant and benign tumors has led to an increase in its indications for pancreatic neoplasms. However, due to the loss of pancreatic parenchyma during surgery, there is a decrease in the functional capacity of the pancreas, leading to EPI. Moreover, surgical resection of the stomach and/or small bowel during pancreatic resection can lead to discoordination of the appropriate production, recycling, and release of pancreatic enzymes termed “postcibal asynchrony” ^12^. The incidence of EPI is highly variable and based on the type of pancreatic resection and its indication. In a recent systematic review following pancreaticoduodenectomy, EPI was observed in 25.2% and 49.1% of patients in benign and malignant tumor groups, respectively ^24^. Comparatively, the incidence of EPI after distal pancreatectomy is within the range of 15–42%, followed by approximately 10% after central pancreatectomy ^25– 27^. In the presence of pre-existing CP, the prevalence of EPI is even higher, reported to be more than 60% after both pancreaticoduodenectomy and distal pancreatectomy ^27, 28^. Additionally, other factors that contribute to EPI are dependent on the type of surgical reconstruction which may alter gastric emptying, bile acid secretion, and intestinal pH that downstream affects the incorporation of pancreatic enzymes with food ^29^.

### Pancreatic cancer

The mechanism of EPI in pancreatic malignancy is related to ductal obstruction and loss of functional parenchyma due to upstream pancreatic atrophy. Similarly, this is also the cause in cases of intraductal papillary mucinous neoplasm, ampullary cancer, and some benign tumors of the pancreas. The highest rate of EPI exists in patients with non-operable pancreatic malignancy with the reported prevalence rates approximately between 50 and 90% ^30^. Expectedly, the rate of EPI in patients who are diagnosed earlier in their disease course and subsequently are deemed surgical candidates have a lower mean prevalence of EPI ranging from 20–44%, rising to over 70% following their surgery ^31^.

### Cystic fibrosis

Cystic fibrosis (CF) is an autosomal recessive disorder characterized by a mutation in the CF transmembrane conductance regulator (CFTR) gene. CFTR in the pancreas is expressed significantly in pancreatic ductal cells responsible for fluid and anion transport into the lumen. Alterations in CFTR function, specifically in CF, lead to a decrease in pancreatic ductal lumen fluid volume and pH. This variation in gene expression has a downstream effect that causes precipitation of proteinaceous secretions, which obstruct and destroy acinar cells ^32^. Although the severity of EPI is highly dependent on the specific genotypic mutation, 85% of CF patients will develop EPI, with the vast majority present at the time of birth ^33^.

### Diabetes

Patients with type 1 or type 2 DM have been shown to have reductions in fecal elastase levels, with some pathologic changes similar to CP, and this has been termed “diabetic exocrine pancreatopathy”. It is not clear if these patients actually have EPI as well. The potential mechanisms of EPI in type 1 and type 2 DM are multifactorial and could include a) exocrine tissue involvement in autoimmune destruction, b) lack of trophic action of insulin on acinar cells, c) microvascular damage to exocrine tissue, and d) effect of enteropancreatic reflex impairment from diabetic neuropathy ^34, 35^. The prevalence of EPI, as documented by fecal elastase, has been reported in one systematic review to be an average of 40% in type 1 DM and 27% in type 2 DM ^36^, although significant heterogeneity exists between reported studies given the lack of exclusion of patients with previous pancreatic disease.

## Consequences and treatment of EPI

Symptoms of EPI develop when approximately 90% of the exocrine function of the pancreas is lost, with manifestations that include steatorrhea (often without diarrhea), abdominal bloating, weight loss, various vitamin deficiencies, and metabolic bone disease. Although patients with EPI may not digest fat, protein, and carbohydrates efficiently, malabsorption of fat appears to be the most clinically impactful. There are a number of reasons for this, including the susceptibility of lipase to inactivation and alternative mechanisms for protein and carbohydrate digestion. Ongoing fat malabsorption leads to nutritional deficiencies of fat-soluble vitamins (A, D, E, and K) along with possible deficiencies of calcium, folic acid, magnesium, thiamine, and zinc. As a result of decreased absorption of vitamin D, patients with EPI are at high risk of developing osteopenia or osteoporosis due to decreased bone mineral density ^37, 38^. The prevalence of overall osteopathy (osteopenia and osteoporosis) is more than 60% in patients with CP, with one in four patients having osteoporosis ^39^. As a result, initial and serial bone mineral density testing should be done in all patients with EPI, along with baseline evaluation of nutritional status and periodic measurements of fat-soluble vitamins ( Table 1).

**Table 1.** Baseline measurements and interventions to consider prior to initiating pancreatic enzyme replacement therapy.

Anthropometrics - Body mass index - Muscle mass or strength ▪ Computed tomography scan of psoas muscle at L3 level ▪ Handgrip strength
Basic nutritional parameters - Albumin, prealbumin, and retinol-binding protein - Vitamin D, A, and E level - International normalized ratio - B vitamins and folate - Hemoglobin A1c
Dual energy X-ray absorptiometry scan
Smoking cessation
Alcohol abstinence
Assessment of lifestyle parameters which might augment the risk of osteoporosis (physical activity)

Treatment of EPI consists of providing activated digestive enzymes to the duodenum during the prandial and post-prandial period. In normal health, most fat and fat-soluble vitamin absorption occurs in the duodenum, with significantly less in the jejunum. The human pancreas in healthy adult subjects is estimated to produce at least 900,000 USP units of lipase with every meal. This amount is actually significantly more than is necessary for relatively normal digestion of fats, and it is estimated approximately 10% of this is required to have adequate digestion of nutrients. This would suggest at least 90,000 USP units of lipase would need to be prescribed with each meal. When prescribing PERT, however, the full 90,000 USP units of lipase may not be required given that many patients retain some residual secretion of lipase from the pancreas. In addition, there may be a compensatory increase in gastric lipase secretion of variable degree, which may also provide some improvement in digestion. Despite these considerations, a minimal starting dosage of at least 40,000 to 50,000 USP units of lipase with each meal and half with snacks is recommended ( Table 2). If there is concern for inadequate response, the dosage can be adjusted to 90,000 USP units with each meal, or more if needed.

**Table 2.** Enzyme products available in the US.

Product	Formulation	Lipase content (USP units)/capsule or pill
Zenpep®	Enteric-coated porcine	3,000, 5,000, 10,000, 15,000, 20,000, 25,000, and 40,000
Creon®	Enteric-coated porcine	3,000, 6,000, 12,000, 24,000, and 36,000
Pancreaze®	Enteric-coated porcine	4,200, 10,500, 16,800, and 21,000
Pertzye®	Enteric-coated porcine with bicarbonate	4,000, 8,000, and 16,000
Viokace®	Non-enteric-coated porcine tablet	10,440 and 20,880

Enzyme supplements need to be administered during the meal. A typical regimen is to divide the pills so that they are taken during both the early and the late portion of the meal (e.g. half of the pills after a few bites, and the rest of the pills as the final few bites are ingested). Clinical effectiveness is best determined by the demonstration of steatorrhea improvement, but this is rarely feasible. Instead, improvements in visible steatorrhea, fat-soluble vitamin levels, nutritional indices (e.g. retinol-binding protein), muscle strength, and quality of life provide surrogate measures of success. In those patients who seem to be failing therapy, the most common indication is non-compliance (sometimes owing to cost), wrong timing of ingestion, or inadequate dosage.

In some individuals, an inadequate response to PERT can also be due to inactivation of lipase by gastric acid. This is particularly an issue when using the non-enteric-coated oral PERT preparation, which requires simultaneous acid-reducing agents to be taken. Some data suggest that acid-reducing agents might even increase the overall efficacy of enteric-coated enzyme formulations, allowing them to be released from their pH-sensitive delivery systems more proximally in the small intestine, where fat absorption normally occurs ^40^. In those who continue to fail PERT therapy and/or lose weight, consideration should be given to alternative diagnoses including small intestinal bacterial overgrowth ^41, 42^, the development of secondary pancreatic or non-pancreatic malignancy, or an unrelated condition such as celiac disease.

## Benefits and application of pancreatic enzyme replacement therapy

In a large meta-analysis by de La Iglesia-Garcia *et al*. ^43^, treatment with PERT in patients with CP improved both fat and protein absorption. Additionally, PERT was seen to improve symptoms of abdominal pain, flatulence, and stool consistency. Although similar results were not seen in a previous Cochrane review ^44^, this may have been because only two relevant studies were included at the time. In the more recent meta-analysis of 14 randomized controlled trials (RCTs), PERT appears to have clear nutritional and symptomatic benefit in patients with EPI.

The therapeutic effects of PERT appear to go beyond the resolution of steatorrhea and weight loss alone. In a large prospective longitudinal cohort study in patients with CP, EPI has been shown to be an independent risk factor for mortality ^45^. Although the impact of PERT was not directly assessed in this study, an analysis of CP patients who were receiving PERT following pancreatic surgery showed improved survival ^45^. Similarly, improved survival outcomes have been shown in multiple large cohort studies in patients using PERT following pancreatic cancer surgery ^46– 48^. Quality of life has also been assessed; however, no long-term RCTs exist for this outcome. Nevertheless, in one open label 51-week extension of a previous double blind RCT of PERT in the treatment of EPI, quality of life was seen to significantly improve from baseline ^49^. Significant data also exist on the benefit of PERT in pancreatic cancer. Of note, one recent population-based analysis demonstrated improved survival in pancreatic cancer patients receiving PERT, in which survival time more than doubled ^50^. This benefit of PERT was equivalent to the survival advantage of receiving chemotherapy or undergoing surgery for pancreatic cancer.

The benefits in treatment of EPI have led to practice guideline recommendations for the use of PERT in patients with CP, pancreatic cancer, and pancreatic surgery ^27^. However, most analyses suggest a majority of patients in these groups are either not treated or treated with inadequate dosages. In a European survey study of patients with EPI due to CP or pancreatic cancer, one-quarter of patients took three or fewer capsules a day and more than two-thirds of patients reported ongoing symptoms of steatorrhea ^51^. Similarly, in a Dutch national survey of patients with CP and EPI, the median initiating daily dosage was suboptimal (four capsules daily, 25,000 USP units of lipase each), and a majority of patients reported ongoing gastrointestinal symptoms of EPI including steatorrhea and weight loss ^52^. More recently, in a US population-based analysis, 6.5% of patients with CP received any testing for EPI, 30.4% received a prescription for PERT, and only 10% received a minimally effective dose ^53^. Likewise, in a similar analysis of patients with pancreatic cancer, only 1.9% of patients had testing for EPI and 22% received a prescription for PERT, with only 5.5% receiving an adequate dose ^54^. Ultimately, these studies highlight that despite the common association of EPI with CP and pancreatic cancer, diagnostic testing is rarely performed, treatment with PERT is infrequent, and, if utilized, the dosage amounts are frequently inadequate.

## Conclusions

EPI is frequently an unrecognized consequence of benign and malignant pancreatic disease, leading to various consequences such as abdominal discomfort, steatorrhea, malnutrition, sarcopenia, osteoporosis, weight loss, reduced quality of life, and diminished survival. Despite these rather severe outcomes, the diagnosis and management of EPI is suboptimal, which underscores the importance of awareness in high-risk patients, appropriate use of diagnostic testing, and understanding of treatment goals and strategies. However, current literature suggests that patients who are at high risk of developing EPI do not undergo testing or are not treated with adequate dosages. This review highlights the need for increased efforts in awareness of EPI and more widespread implementation of effective diagnoses and treatment optimization when managing patients with PERT. 
